# Soil storage temperature and air-drying did not significantly change bacterial taxa in the short-term

**DOI:** 10.7717/peerj.20162

**Published:** 2025-10-06

**Authors:** Mingming Du, Peipei Xue, Budiman Minasny

**Affiliations:** Sydney Institute of Agriculture, The University of Sydney, NSW, Australia

**Keywords:** Soil bacteria, Soil storage, Abundant and rare species

## Abstract

Analysing soil microbial communities is vital for understanding ecosystem health, but samples from remote locations often require preservation before DNA can be extracted. In this study, we used the 16S rRNA amplicon sequencing to investigate how different storage methods affect soil microbial diversity over 4 weeks. We implemented storage temperature and moisture as two experimental factors. Three topsoils (sandy, silty loam, and clay loam) were collected and stored under four conditions: frozen at −20 °C, refrigerated at 4 °C, room temperature, and air-dried. DNA extractions and sequencing were performed at 1, 3, 7, 14, and 28 days. Our results revealed that storage temperature and moisture did not substantially change bacterial diversity and composition across all three soil types. Rare taxa showed a similar pattern to abundant taxa but were more variable in beta diversity. Overall, all tested methods effectively preserved both abundant and rare taxa in the short term. However, the long-term impacts of the sample storage, particularly on rare taxa, need further exploration.

## Introduction

Soil microbes rank among the most diverse communities on Earth, playing essential roles in ecosystem function and housing the planet’s genetic diversity (Wagg et al., 2019). Technological advances in DNA sequencing have enabled soil scientists to explore the soil microbiome (Fierer, 2017). Ideally, soil scientists should extract DNA immediately after sampling. However, the practical challenges of remote fieldwork and transit delays often make this difficult. Therefore, it is crucial to understand how the microbial communities respond to the different storage methods to find the optimal way to store them.

Two crucial sample processing factors (a) temperature and (b) moisture, may alter experimental results. However, previous studies have investigated that storage temperatures slightly alter microbial community structures in the short term, as indicated by shifts in bacterial diversity (Edwards et al., 2024; Lauber et al., 2010; Rubin et al., 2013). Besides temperature, water loss may also trigger the microbial community to shift since soil moisture directly regulates microbes metabolic functions and indirectly affects nutrient availability (Schimel, 2018). For example, soil moisture content can shape microbial community dynamics as aridity slows DNA degradation (Brockett, Prescott & Grayston, 2012; Sirois & Buckley, 2019). Besides, the storage time also shapes bacterial diversity, with a decline in Shannon diversity observed after 6 weeks (Kushwaha et al., 2024). Previous research has produced conflicting results on the impact of air-drying storage methods on soil microbial communities (Lane et al., 2022; Qiu et al., 2020; Wang et al., 2021). For instance, Lane et al. (2022) found a pronounced influence of air drying on β-diversity, while Wang et al. (2021) detected minimal impact on microbial composition. These inconsistency among studies may result from variations in climatic conditions, soil characteristics, and methodological design, highlighting the ongoing challenge of finding a consistently reliable preservation strategy.

In most soil ecosystems, the microbial community is uneven distributed, with a few abundant taxa and a large number of rare taxa (Jousset et al., 2017; Nemergut et al., 2011). The abundant taxa are known for their high prevalence and key function in processes like carbon cycling (Wu et al., 2017). The rare taxa contribute unique functional traits and acting as a dormant “seed bank” that can enhance ecosystem resilience to environmental changes (Jousset et al., 2017; Lennon & Jones, 2011). However, most studies on soil sample storage have focused only on the total community structure, overlooking the different responses of abundant and rare microbes (Delavaux et al., 2020; Lane et al., 2022; Rubin et al., 2013; Wang et al., 2021). Yet, emerging studies illuminated the ecological significance of abundant and rare taxa (Jiao et al., 2019; Lynch & Neufeld, 2015). Therefore, it is essential to clarify how soil storage methods affect the temporal dynamics of abundant and rare soil microbes.

To explore the influences of storage conditions on soil microbial communities, we conducted a 1-month investigation to elucidate the effects of four distinct soil storage conditions (20 °C, 4 °C, and room temperature (approximately 20 °C), and air-drying) on bacterial communities across three soil types. Using 16S rRNA sequencing, we aimed to address the following research questions: (i) How do storage conditions affect soil diversity and composition? (ii) Does the impact of storage conditions vary in the different soil types? (iii) Do rare taxa and abundant taxa show a similar response to storage-induced changes?

## Materials and Methods

### Soil sampling

Soil samples for this study were collected in June 2023 from Camden farm, Sydney, NSW, Australia. The site is managed under a wheat cultivation system. Within a 1 km radius, soil samples representing three soil textures—sandy, silty loam, and clay loam—were collected (Schad, 2023). The sample collection was conducted using a sterilised hand trowel, disinfected with 80% alcohol before each use. About 1 kg of topsoil (0–10 cm depth) was collected at each site, subsequently stored in transparent, sealed bags, and transported to the laboratory, 1 h’s drive away. Bulk soil was collected, and visible plant tissues and roots were removed. Upon laboratory arrival, samples were homogenised and subset into four subsamples for the different storage treatments: frozen at −20 °C, refrigerated at 4 °C, room temperature, and air-drying. For the room temperature samples, the samples were sealed with Parafilm to maintain the moisture. DNA was extracted on days 1, 3, 7, 14, and 28, except for samples frozen at −20 °C, which were extracted only on days 1 and 28.

The fundamental physical and chemical soil properties were quantified following our previous protocol (Du, Minasny & Rabbi, 2024). Total carbon and total nitrogen were determined *via* dry combustion using a LECO CN analyser (LECO Corporation, St. Joseph, MI, USA). Electrical conductivity was assessed using a 1:5 soil-to-deionized water ratio. Soil pH was measured with 25 ml of 0.01 M CaCl_2_ solution. Additionally, soil particle size distribution was analysed using the hydrometer method. As summarised in Table 1, silty loam exhibited the highest TC (2.47%), exceeding sandy (1.47%) and clay loam (1.22%) soils. Clay loam showed the greatest EC and pH (266 µS cm^−1^ and 6.26, respectively).

**Table 1:** Soil basic properties across three soil types.

	C%	N%	Clay%	Silt%	Sand%	EC (μS/cm)	pH (CaCl_2_)
Sandy	1.47	0.15	18.2	8.1	73.8	93.3	5.58
Silty loam	2.47	0.22	21.3	25.5	53.2	68.5	5.37
Clay loam	1.22	0.12	29.3	16.7	53.9	266	6.26

### Soil DNA sequencing and analysis

Soil DNA was extracted using Qiagen DNeasy PowerSoil Pro Kit (Qiagen, Hilden, Germany) following the manufacturer’s standard procedures. The purified DNA was assessed for yield and quality using the Quantifluor dsDNA system. Three replicate DNA samples were extracted per soil sample, and these replicates were pooled prior to sequencing. Metabarcoding of bacterial communities was conducted using primers 341F and 805R (Takahashi et al., 2014). A two-step PCR protocol was used to generate dual-indexed amplicons adapted from the Illumina protocol for 16S Metagenomic Sequencing Library Preparation. The concentration of PCR amplicons was then measured by fluorimetry using the Quantifluor dsDNA system. Amplicons were then pooled at equimolar concentrations, purified using SPRI beads normalised to a concentration of 10 nM and sequenced by the IMB Sequencing Facility at the University of Queensland on an Illumina MiSeq (2 × 300 bp).

### Bioinformatics

The bioinformatics workflow was carried out using the LatchBio workflow. Cutadapt was used to remove primer sequences for pair-end reads (Martin, 2011). All subsequent processing and analysis were carried out in R version 4.0 (R Core Team, 2020). Amplicon sequence variants (ASVs) and taxonomic inference were conducted through DADA2 package (Callahan et al., 2016a). During quality filtering in DADA2, forward and reverse reads were truncated to lengths of 270 and 240 bp, respectively. Following truncation, reads exceeding maximum expected error thresholds of 2 (forward) and 3 (reverse) were also discarded. The ‘Pseudo Pooling’ method was used for the main ‘dada’ function. Chimeras were identified and removed with the ‘consensus’ method of the “removeBimeraDenovo” function. The naïve Bayesian Classifier was used to assign taxonomy to genus level with the reference to Silva 138 database (Quast et al., 2012) using the “assignTaxonomy” function of DADA2.

We filtered out ASVs detected with fewer than 15 reads across all samples and found in fewer than two samples, minimising potential biases (Du et al., 2025). Then, the ASVs were rarefied to the minimum reads (33,000 reads) to standardise the bacterial community. The distinction of taxa as abundant or rare was determined by their relative abundance. Specifically, ASVs exhibiting a relative abundance exceeding 0.1% were classified as abundant taxa, whereas those below 0.01% were identified as rare taxa (Zheng et al., 2021).

### Statistical analysis

Using the ‘vegan’ package (Oksanen et al., 2013), we analysed various alpha diversity indices to discern the impacts of storage methods and duration on bacterial diversity. A linear mixed-effects model with random effects including ‘soil types’ was applied to evaluate the effect of storage methods and time on microbial alpha diversity by the ‘lme4’ package (Bates et al., 2015). We created the phylogenetic tree by ‘phyloseq’ package (Callahan et al., 2016b) and calculated the weighted UniFrac distance for the principal coordinate analysis (PCoA) to show bacterial community differences between storage methods and time by the ‘microeco’ package (Liu et al., 2021). Additionally, a permutational multivariate analysis of variance (PERMANOVA) was conducted to quantify the variances in bacterial distribution due to storage methods and time. All data visualisations were performed using R Studio (version 4.3.2).

## Results

### Alpha diversity

**Figure 1:**
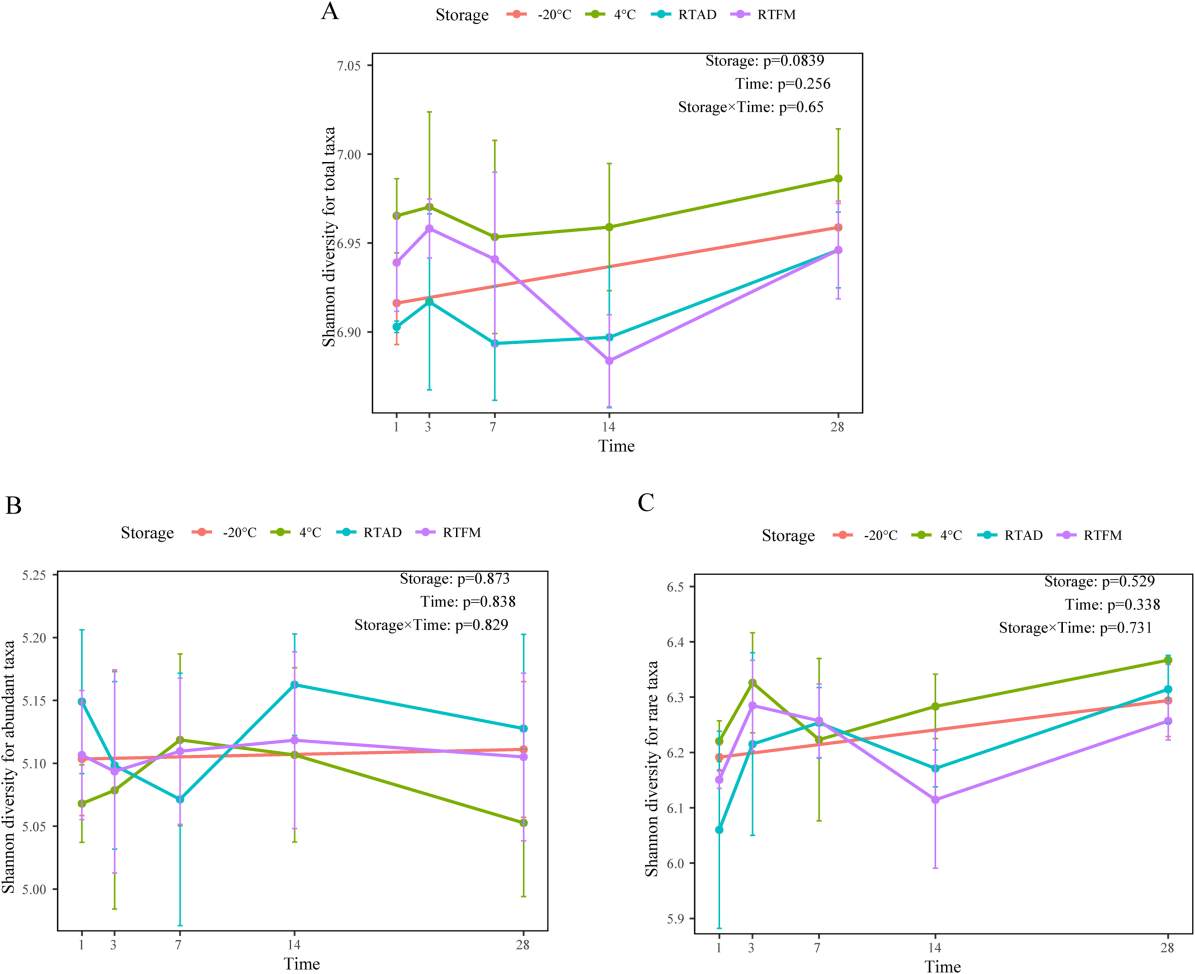
The temporal change of four storage methods on Shannon diversity. (A) Total taxa. (B) Abundant taxa. (C) Rare taxa.

Through 16S rRNA sequencing, 6,005 ASVs (33,000 sequences per sample after rarefication) were generated in the soil bacterial community analysis. Across the 1-month storage experiment, we did not find a significant difference among the storage methods over time in terms of four alpha diversity metrics for total, abundant, and rare taxa (Table 2). For the total taxa, the mean Shannon index was slightly higher under 4 °C storage compared to the other conditions (Fig. 1A). However, this trend was not consistent across all soil types. Specifically, the increase was observed in silty loam and sandy soil but was absent in the clay loam (Fig. S1). For the abundant and rare species, the Shannon index fluctuated slightly but remained consistent throughout the storage period (Figs. 1B and 1C).

**Table 2:** ANOVA table for the effects of different storage methods, time, and their respective interactions.

	Factor	Df	F-value			
			ACE	Chao1	Observed	Shannon
Total taxa	Storage	3	0.777	0.791	0.765	1.421
	Time	4	1.077	1.051	0.957	0.686
	Storage × Time	9	0.096	0.106	0.094	0.100
Abundant taxa	Storage	3	/	/	0.367	0.129
	Time	4	/	/	1.007	0.402
	Storage × Time	9	/	/	0.256	0.171
Rara taxa	Storage	3	0.568	0.711	0.471	0.420
	Time	4	1.862	1.779	1.910	1.761
	Storage × Time	9	0.109	0.100	0.112	0.185

**Note:**

The ‘/’ indicates that statistical analysis was not performed. No significant differences were observed.

### Beta diversity

**Figure 2:**
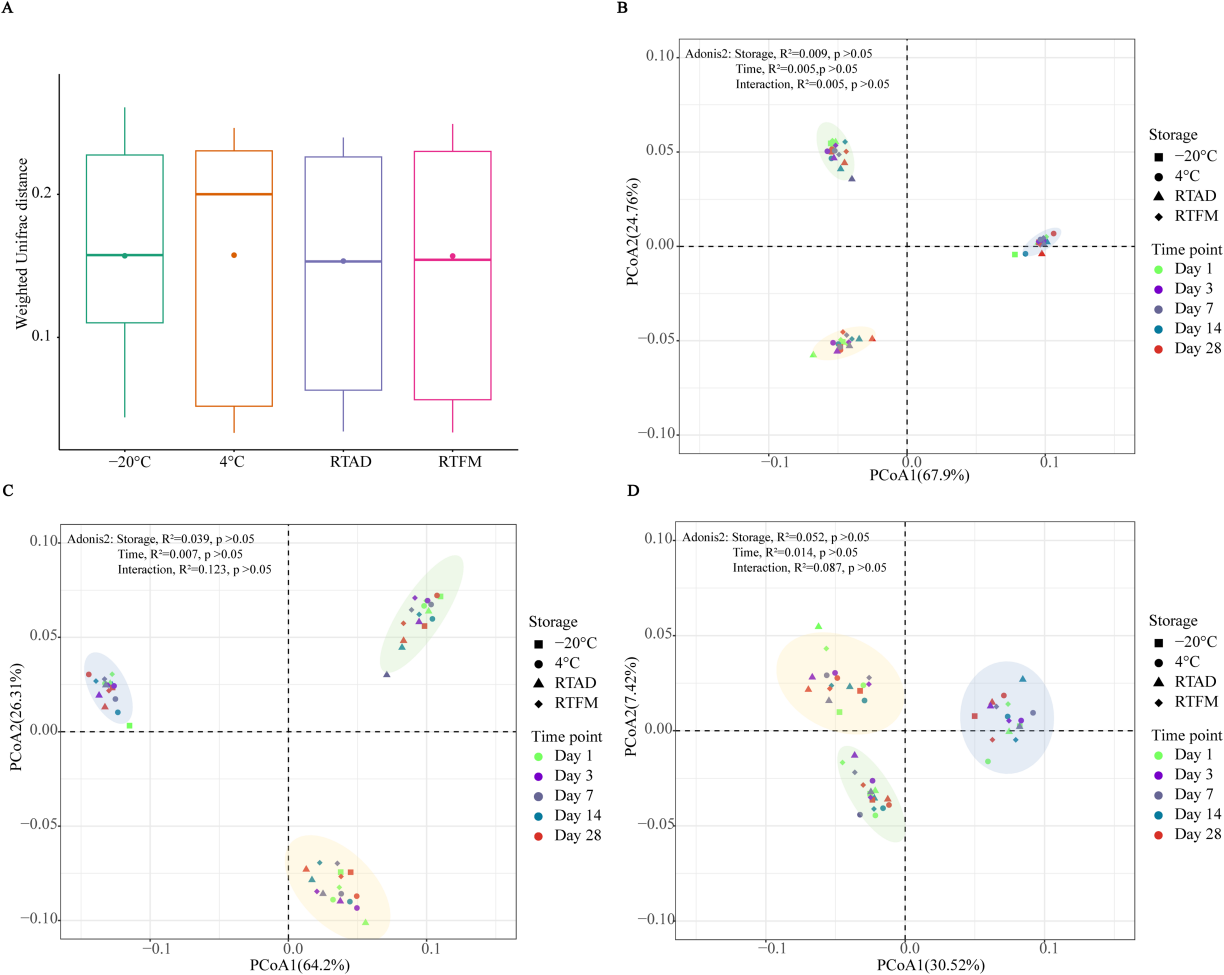
Bacterial diversity differences across four storage methods. (A) Weighted UniFrac distances for total taxa across the four storage conditions; no statistically significant differences were observed. (B–D) Principal coordinates analysis (PCoA) of weighted UniFrac distances for (B) total, (C) abundant, and (D) rare taxa. Ellipses are coloured by soil type—silt loam, sandy, and clay loam—shown in yellow, green, and blue, respectively. Storage conditions were 4 °C, −20 °C, room temperature with air-drying (RTAD), and room temperature with field moisture (RTFM). Effects of time and storage condition on community dissimilarity were tested using PERMANOVA (adonis2).

Based on Weighted UniFrac distances, the four storage treatments did not result in a significant difference in the beta diversity of the total taxa (Fig. 2A). The bacterial community composition also didn’t show significant differences between storage methods and time for the total (Fig. 2B), abundant (Fig. 2C) and rare taxa (Fig. 2D). In the principal coordinate analysis (PCoA), the first two axes explained a substantial portion of the variance for both the total (67.9% and 24.76%) and abundant communities (64.2% and 26.31%). In contrast, the variance explained was considerably lower for the rare taxa (30.52% and 7.42%). Notably, the groups of three soil types were clearly clustered, across total, abundant, and rare taxa, indicating that the four preservation methods effectively maintained the general beta diversity of each soil type.

### Community composition

Despite initial differences between the three soil types, their microbial community compositions at the phylum level remained stable over time across all storage methods. This stability was observed for the total (Fig. S2), abundant (Fig. S3), and rare taxa (Fig. S4). Across all communities, Actinobacteria, Proteobacteria, and Acidobacteria were the three most abundant phyla. We also noted a minor decrease in the relative abundance of Acidobacteria among the abundant taxa in the air-dried silty loam and the sandy soil stored at room temperature with field moisture.

## Discussion

Our 1-month study demonstrated that different storage methods had limited effects on microbial diversity and community composition across three different soil types. All tested storage methods, regardless of temperature or moisture, successfully preserved both α- and β-diversity. This indicates that researchers can be confident in these common preservation techniques for short-term storage.

Notably, we found that air-drying did not significantly change the bacterial diversity (Figs. 1 and 2), aligns with previous research showing it is an effective preservation method for soils from various environments, including fluvial plains, croplands, and woodlands (Ivanova et al., 2017; Martí et al., 2012; Smenderovac et al., 2024; Wang et al., 2021). This resilience could stem from bacterial evolved mechanisms for coping with water loss. For instance, bacteria may enter a state of dormancy in the absence of water, which slows DNA degradation and helps stabilise the microbial community composition (Laskowska & Kuczyńska-Wiśnik, 2020).

Although the overall diversity remained stable, we did observe fluctuations in the Shannon diversity during the storage time especially for the sandy and silty loam soils in 4 °C (Fig. 1). These may be attributed to the varied micro-habitats resulting from the physical heterogeneity of the soil (Lehmann et al., 2008). The environmental characteristics and resource availability of the micro-habitat might also contribute to the observed minor variability among bacterial community composition at the phylum level (Fig. S2). This is expected, as a single gram of soil can encompass up to 10^6^ unique bacterial and archaeal taxa (Gans, Wolinsky & Dunbar, 2005). Previous study using single soil samples showed little repetition by the SSU rRNA sequences obtained (Zhou et al., 2004). Therefore, the small variation we observed emphasized the challenge of achieving complete homogenization of soil microbial communities.

A key finding of this study was the different response of rare and abundant taxa to storage. While rare taxa clustered by the soil type, they exhibited greater dispersal on the PCoA plots compared to their abundant counterparts (Fig. 2). This pattern suggests that rare taxa exhibited reduced phylogenetic relatedness among the storage conditions, indicating that some rare taxa may be particularly vulnerable to temperature or moisture changes. Such sensitivity may be associated with the dynamic interactions within bacterial communities over time, which can drive shifts in community structure and foster divergence (Rubin et al., 2013). It is also important to consider the technological limitations, such as sequencing errors, can make the detection and analysis of rare taxa more challenging (Reeder & Knight, 2009). Despite this variability, the changes were not significant to alter the bacterial diversity and composition over the 1-month period.

It is important to acknowledge the limitations of our study. Our soil samples were collected from a humid subtropical climate in Australia. Microbial communities in other climatic zones, such as the tropics or tundra, might respond differently to these preservation methods. Furthermore, given that our experiment lasted only 1 month, the long-term effects of storage on microbial diversity, especially for rare taxa, require further exploration.

## Conclusion

Our study confirms that the tested storage methods (freezing, refrigeration, room temperature, and air-drying) effectively preserve the diversity and composition of the bacteria abundant and rare taxa for up to a month. We hope that the information we have provided here about the effectiveness of existing soil storage methods can help researchers choose optimal preservation conditions for soil samples, thereby ensuring the accuracy of microbial community analyses. Future researchers should also test the effect of storage on different soil types to evaluate how long the bacterial diversity and composition will remain stable.

## Supplemental Information

10.7717/peerj.20162/supp-1 Supplemental Information 1 Supplementary Figures.

## Additional Information and Declarations

### DNA Deposition

The following information was supplied regarding the deposition of DNA sequences:

The sequence reads are available at GenBank: PRJNA1281203.

For review: https://figshare.com/articles/dataset/USYD-06-2023_1_zip/29539181?file=56170367.

### Data Availability

The following information was supplied regarding data availability:

The raw data is available at Zenodo: Mingming-Du. (2025). Mingming-Du/Decay-experiment: Decay experiment-first release (V1.0.0). Zenodo. https://doi.org/10.5281/zenodo.17041991.

## References

[ref-1] Bates D, Mächler M, Bolker B, Walker S (2015). Fitting linear mixed-effects models using lme4. Journal of Statistical Software.

[ref-2] Brockett BF, Prescott CE, Grayston SJ (2012). Soil moisture is the major factor influencing microbial community structure and enzyme activities across seven biogeoclimatic zones in western Canada. Soil Biology and Biochemistry.

[ref-3] Callahan BJ, McMurdie PJ, Rosen MJ, Han AW, Johnson AJA, Holmes SP (2016a). DADA2: high-resolution sample inference from Illumina amplicon data. Nature Methods.

[ref-4] Callahan BJ, Sankaran K, Fukuyama JA, McMurdie PJ, Holmes SP (2016b). Bioconductor workflow for microbiome data analysis: from raw reads to community analyses. F1000Research.

[ref-5] Delavaux CS, Bever JD, Karppinen EM, Bainard LD (2020). Keeping it cool: soil sample cold pack storage and DNA shipment up to 1 month does not impact metabarcoding results. Ecology and Evolution.

[ref-6] Du M, Minasny B, Rabbi SM (2024). Carbon to nitrogen stoichiometry of organic amendments influences the improvement of aggregate stability of a cropping vertisol. Soil Use and Management.

[ref-7] Du M, Xue P, Minasny B, McBratney A, Tang Y (2025). Patterns, processes, and predictions: soil bacteria unique habitats along a megametre transect in Eastern Australia. Soil Ecology Letters.

[ref-8] Edwards JD, Love SJ, Phillips RP, Fei S, Domke G, Parker JD, McCormick M, LaRue EA, Schweitzer JA, Bailey JK (2024). Long-and short-term soil storage methods other than freezing can be useful for DNA-based microbial community analysis. Soil Biology and Biochemistry.

[ref-9] Fierer N (2017). Embracing the unknown: disentangling the complexities of the soil microbiome. Nature Reviews Microbiology.

[ref-10] Gans J, Wolinsky M, Dunbar J (2005). Computational improvements reveal great bacterial diversity and high metal toxicity in soil. Science.

[ref-11] Ivanova EA, Korvigo IO, Aparin BF, Chirak EL, Pershina EV, Romaschenko NS, Provorov NA, Andronov EE (2017). The preservation of microbial DNA in archived soils of various genetic types. PLOS ONE.

[ref-12] Jiao S, Wang J, Wei G, Chen W, Lu Y (2019). Dominant role of abundant rather than rare bacterial taxa in maintaining agro-soil microbiomes under environmental disturbances. Chemosphere.

[ref-13] Jousset A, Bienhold C, Chatzinotas A, Gallien L, Gobet A, Kurm V, Küsel K, Rillig MC, Rivett DW, Salles JF (2017). Where less may be more: how the rare biosphere pulls ecosystems strings. The ISME Journal.

[ref-14] Kushwaha P, Soto Velázquez AL, McMahan C, Neilson JW (2024). Field to greenhouse: how stable is the soil microbiome after removal from the field?. Microorganisms.

[ref-15] Lane JM, Delavaux CS, Van Koppen L, Lu P, Cade-Menun BJ, Tremblay J, Bainard LD (2022). Soil sample storage conditions impact extracellular enzyme activity and bacterial amplicon diversity metrics in a semi-arid ecosystem. Soil Biology and Biochemistry.

[ref-16] Laskowska E, Kuczyńska-Wiśnik D (2020). New insight into the mechanisms protecting bacteria during desiccation. Current Genetics.

[ref-17] Lauber CL, Zhou N, Gordon JI, Knight R, Fierer N (2010). Effect of storage conditions on the assessment of bacterial community structure in soil and human-associated samples. FEMS Microbiology Letters.

[ref-18] Lehmann J, Solomon D, Kinyangi J, Dathe L, Wirick S, Jacobsen C (2008). Spatial complexity of soil organic matter forms at nanometre scales. Nature Geoscience.

[ref-19] Lennon JT, Jones SE (2011). Microbial seed banks: the ecological and evolutionary implications of dormancy. Nature Reviews Microbiology.

[ref-20] Liu C, Cui Y, Li X, Yao M (2021). microeco: an R package for data mining in microbial community ecology. FEMS Microbiology Ecology.

[ref-21] Lynch MD, Neufeld JD (2015). Ecology and exploration of the rare biosphere. Nature Reviews Microbiology.

[ref-22] Martin M (2011). Cutadapt removes adapter sequences from high-throughput sequencing reads. EMBnet. Journal.

[ref-23] Martí E, Càliz J, Montserrat G, Garau MA, Cruañas R, Vila X, Sierra J (2012). Air-drying, cooling and freezing for soil sample storage affects the activity and the microbial communities from two Mediterranean soils. Geomicrobiology Journal.

[ref-24] Nemergut DR, Costello EK, Hamady M, Lozupone C, Jiang L, Schmidt SK, Fierer N, Townsend AR, Cleveland CC, Stanish L (2011). Global patterns in the biogeography of bacterial taxa. Environmental Microbiology.

[ref-25] Oksanen J, Blanchet FG, Kindt R, Legendre P, Minchin PR, O’hara R, Simpson GL, Solymos P, Stevens MHH, Wagner H (2013). Package ‘vegan’. Community Ecology Package, Version.

[ref-26] Qiu Z, Wang J, Delgado-Baquerizo M, Trivedi P, Egidi E, Chen Y-M, Zhang H, Singh BK (2020). Plant microbiomes: do different preservation approaches and primer sets alter our capacity to assess microbial diversity and community composition?. Frontiers in Plant Science.

[ref-27] Quast C, Pruesse E, Yilmaz P, Gerken J, Schweer T, Yarza P, Peplies J, Glöckner FO (2012). The SILVA ribosomal RNA gene database project: improved data processing and web-based tools. Nucleic Acids Research.

[ref-41] R Core Team (2020). R: a language and environment for statistical computing. R Foundation for Statistical Computing. https://www.R-project.org/.

[ref-28] Reeder J, Knight R (2009). The ‘rare biosphere’: a reality check. Nature Methods.

[ref-29] Rubin BE, Gibbons SM, Kennedy S, Hampton-Marcell J, Owens S, Gilbert JA (2013). Investigating the impact of storage conditions on microbial community composition in soil samples. PLOS ONE.

[ref-30] Schad P (2023). World reference base for soil resources—its fourth edition and its history. Journal of Plant Nutrition and Soil Science.

[ref-31] Schimel JP (2018). Life in dry soils: effects of drought on soil microbial communities and processes. Annual Review of Ecology, Evolution, and Systematics.

[ref-32] Sirois SH, Buckley DH (2019). Factors governing extracellular DNA degradation dynamics in soil. Environmental Microbiology Reports.

[ref-33] Smenderovac E, Emilson C, Rheault K, Brazeau É, Morency M-J, Gagné P, Venier L, Martineau C (2024). Drying as an effective method to store soil samples for DNA-based microbial community analyses: a comparative study. Scientific Reports.

[ref-34] Takahashi S, Tomita J, Nishioka K, Hisada T, Nishijima M (2014). Development of a prokaryotic universal primer for simultaneous analysis of Bacteria and Archaea using next-generation sequencing. PLOS ONE.

[ref-35] Wagg C, Schlaeppi K, Banerjee S, Kuramae EE, van der Heijden MG (2019). Fungal-bacterial diversity and microbiome complexity predict ecosystem functioning. Nature Communications.

[ref-36] Wang F, Che R, Deng Y, Wu Y, Tang L, Xu Z, Wang W, Liu H, Cui X (2021). Air-drying and long time preservation of soil do not significantly impact microbial community composition and structure. Soil Biology and Biochemistry.

[ref-37] Wu W, Logares R, Huang B, Hsieh C (2017). Abundant and rare picoeukaryotic sub-communities present contrasting patterns in the epipelagic waters of marginal seas in the northwestern Pacific Ocean. Environmental Microbiology.

[ref-38] Zheng W, Zhao Z, Lv F, Wang R, Wang Z, Zhao Z, Li Z, Zhai B (2021). Assembly of abundant and rare bacterial and fungal sub-communities in different soil aggregate sizes in an apple orchard treated with cover crop and fertilizer. Soil Biology and Biochemistry.

[ref-39] Zhou J, Xia B, Huang H, Palumbo AV, Tiedje JM (2004). Microbial diversity and heterogeneity in sandy subsurface soils. Applied and Environmental Microbiology.

