# Supplementary material for: Soil storage temperature and air-drying did not significantly change bacterial taxa in the short-term
Source: PeerJ. 2025 Oct 6;13:e20162. doi: 10.7717/peerj.20162 (PMC12510243; doi:10.7717/peerj.20162)
Supplement: Supplemental Information 1 [file peerj-13-20162-s001.docx]

### Soil storage temperature and air-drying did not significantly change bacterial taxa in the short-term

Mingming Du^a,b *^, Peipei Xue^a,b^, Budiman Minasny^a,b^

^a^School of Life and Environmental Sciences, The University of Sydney, NSW 2015, Australia

^b^Sydney Institute of Agriculture, The University of Sydney, NSW 2006, Australia

**Corresponding author:* [*mingming.du@sydney.edu.au*](mailto:mingming.du@sydney.edu.au)

**Supplementary materials:**


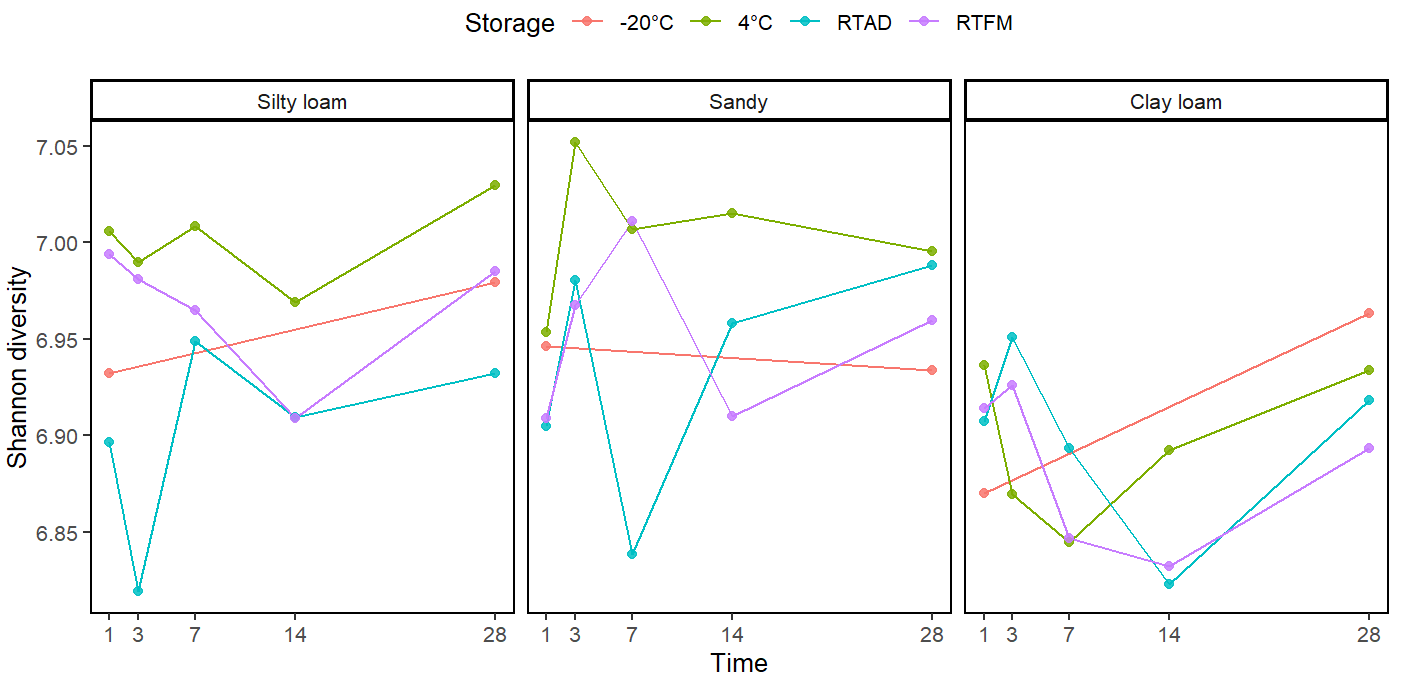


Fig.S1 The Shannon diversity for each soil type under different storage methods


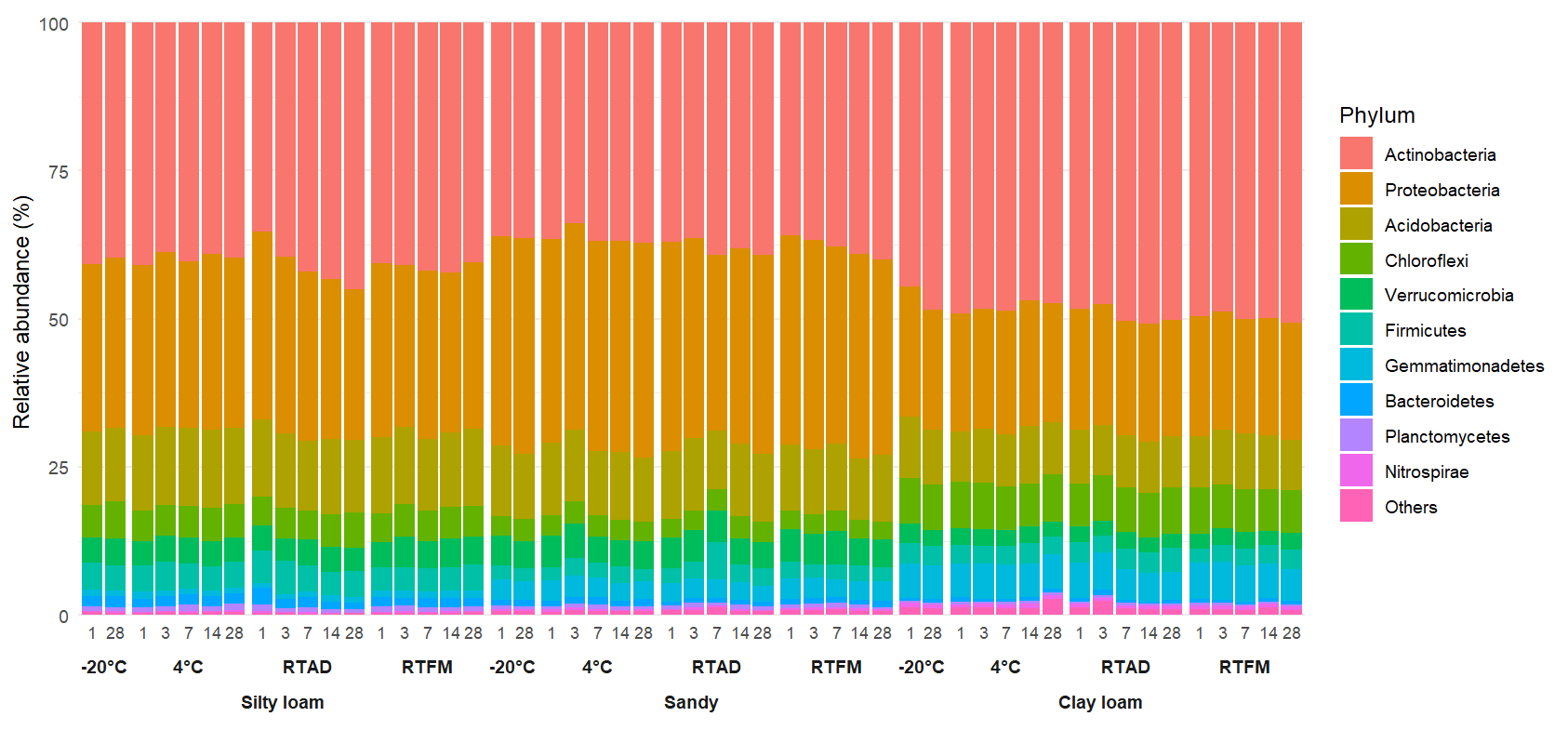


Fig.S2. The relative abundance of the total taxa at the phylum level for each soil type under different storage methods


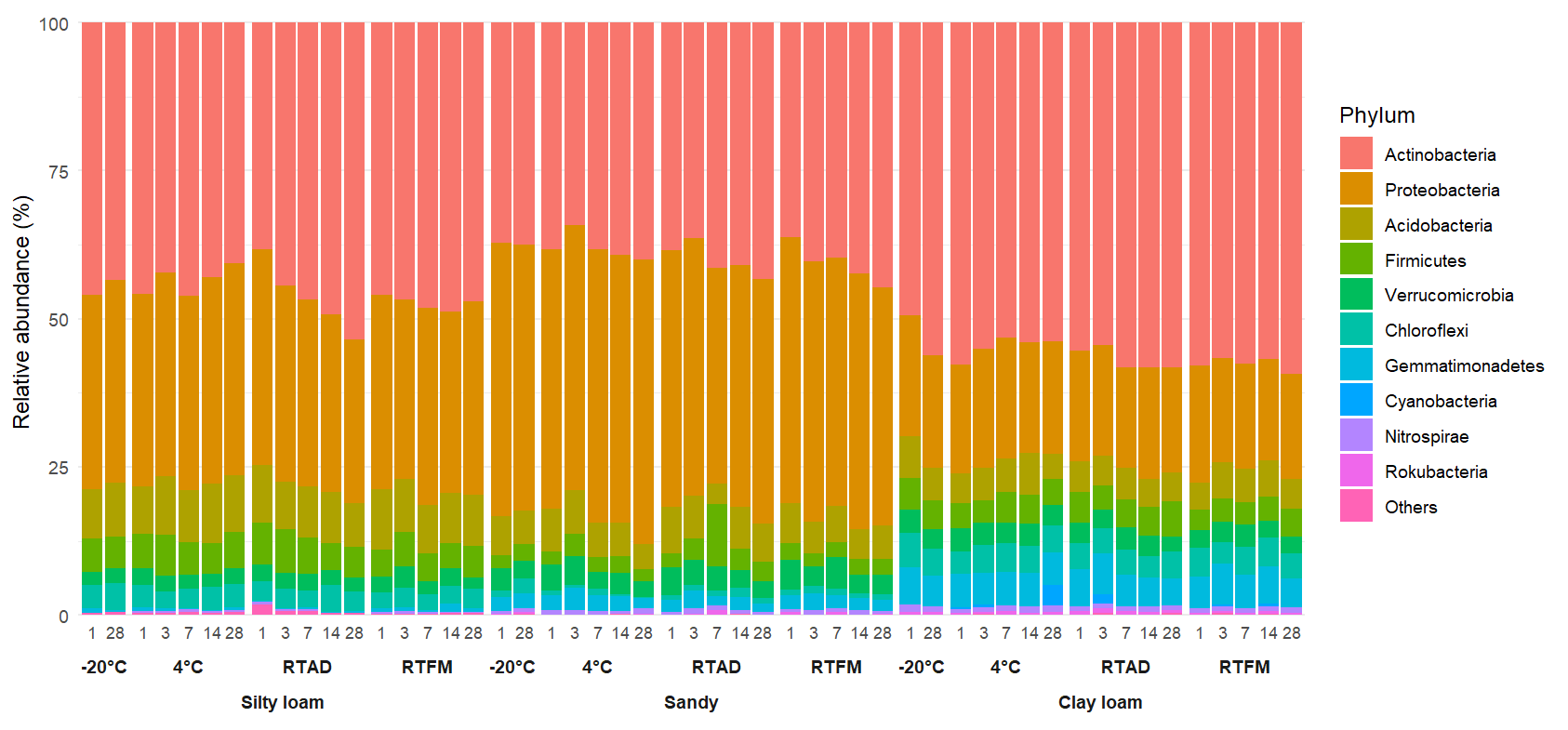


Fig.S3. The relative abundance of the abundant taxa at the phylum level for each soil type under different storage methods


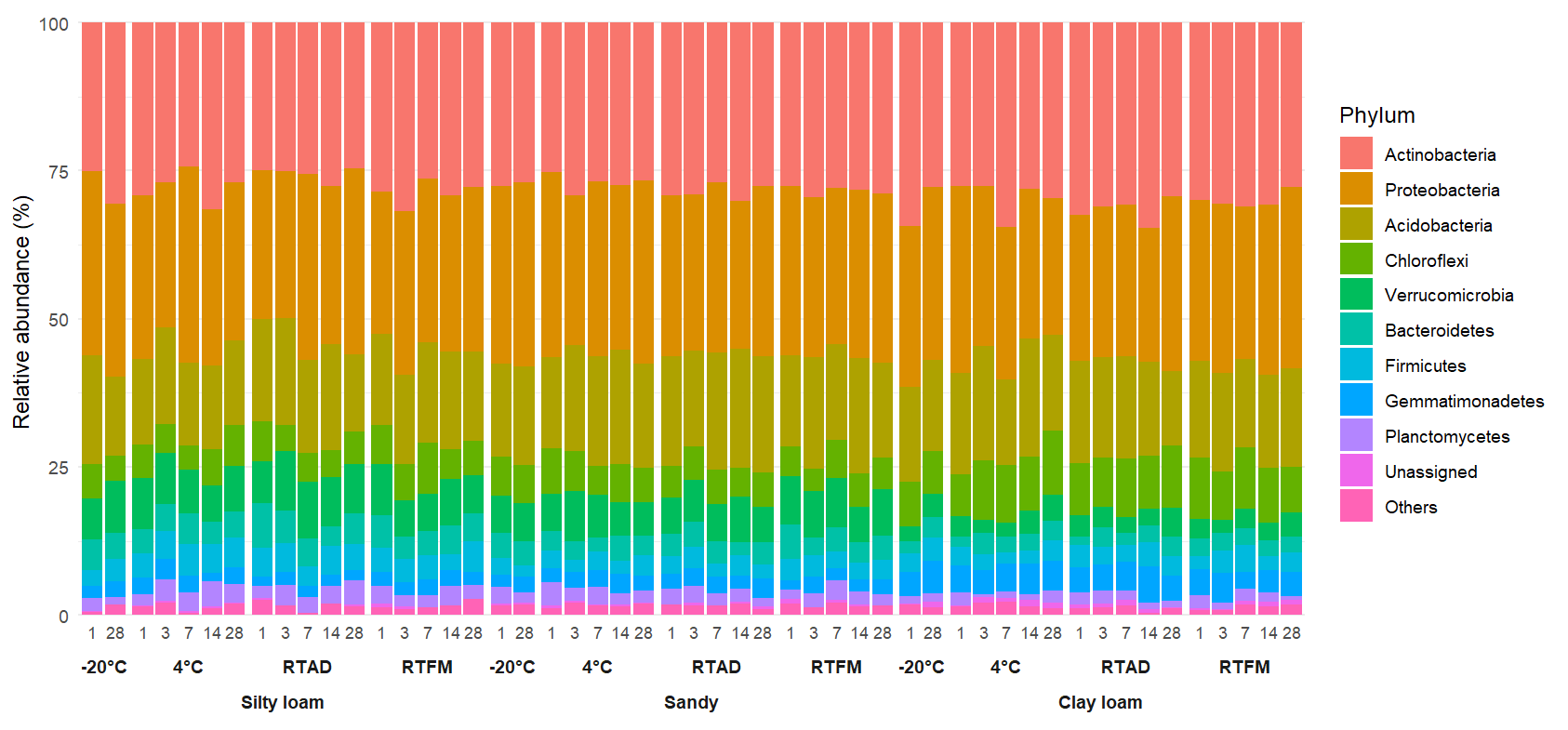


Fig.S4. The relative abundance of the rare taxa at the phylum level for each soil type under different storage methods
